# Mitochondrial Transplantation Moderately Ameliorates Retinal Degeneration in Royal College of Surgeons Rats

**DOI:** 10.3390/biomedicines10112883

**Published:** 2022-11-10

**Authors:** Shih-Fang Wu, Chih-Yao Lin, Rong-Kung Tsai, Yao-Tseng Wen, Feng-Huei Lin, Chia-Yu Chang, Ching-I Shen, Shinn-Zong Lin, Horng-Jyh Harn, Tzyy-Wen Chiou, Chin-San Liu, Yan-Ting Chen, Hong-Lin Su

**Affiliations:** 1 The Joint Ph.D. Program in Tissue Engineering and Regenerative Medicine, National Health Research Institutes and National Chung Hsing University, Taichung 402, Taiwan; 2 Department of Life Sciences, National Chung Hsing University, Taichung 402, Taiwan; 3 Institute of Eye Research, Buddhist Tzu Chi Hospital, Hualien 970, Taiwan; 4 Institute of Biomedical Engineering and Nanomedicine, National Health Research Institutes, Miaoli 350, Taiwan; 5 Bioinnovation Center, Buddhist Tzu Chi Medical Foundation, Hualien 970, Taiwan; 6 Duogenic Stem Cells Corporation, Taichung 402, Taiwan; 7 Department of Neurosurgery, Buddhist Tzu Chi Medical Foundation, Hualien 970, Taiwan; 8 Department of Pathology, Buddhist Tzu Chi Medical Foundation, Hualien 970, Taiwan; 9 Department of Life Science and Graduate Institute of Biotechnology, National Dong Hwa University, Hualien 974, Taiwan; 10 Vascular and Genomic Center, Changhua Christian Hospital, Changhua 500, Taiwan; 11 Departments of Neurology, Changhua Christian Hospital, Changhua 500, Taiwan; 12 Departments of Ophthalmology, Changhua Christian Hospital, Changhua 500, Taiwan

**Keywords:** mitochondrial transplantation, retinal degeneration, retinitis pigmentosa, macular degeneration

## Abstract

Retinal pigmented epithelial (RPE) cells possess high mitochondria content for energy production, which is required for phagocytosis and vision cycle metabolism. The mitochondrial integrity in RPE cells helps the homeostasis of photoreceptor turnover and prevents retina aging and degeneration. Mitochondrial transplantation benefits the recovery of several acute inflammatory diseases, leading us to investigate the effects of mitochondrial transplantation on retina degeneration. Allogeneic mitochondria were isolated and delivered into the vitreous chamber in the Royal College of Surgeons (RCS) rats, which exhibit inherited and early-onset retina degeneration. The progress of retina degeneration was examined with optical coherence tomography (OCT) and visual evoked potential (VEP) to determine the retina thickness and integrity of afferent electrical signals from affected eyes, respectively. We found that mitochondria engraftment moderately attenuated the degeneration of retinal layers in RCS rats by histological examination. This result was consistent with the OCT measurement of retina thickness around the optic disc. The VEP analysis revealed that the peak one (N1) latency, representing the arriving time of electrical impulse from the retina to cortex, was substantially maintained as the normal value after the mitochondrial transplantation. This result suggests that the intra-vitreous transplanted mitochondria ameliorate the degeneration of photoreceptors in RCS rats and might be potential for clinical application.

## 1. Introduction

Retinal degeneration is a global threat to irreversible visual loss in the elderly. Etiologies of retinal degeneration include hereditary genetic defects, the aging process, photodamage, toxicity, etc. Retinitis pigmentosa (RP), a congenital retinal degeneration, mainly affects the young population; in contrast, age-related macular degeneration (AMD) is a leading cause of blindness in the elderly. Research evidence indicates that mitochondrial dysfunction contributes to retina degeneration of both RP and AMD [1,2]

In photoreceptors cells, the localization of the mitochondria is most restricted in the dendrite and inner segment (IS) discs. The outer segment (OS) discs of the photoreceptor cells are daily shed and phagocytized by RPE cells. This phagocytosis requires active mitochondria to produce high contents of ATP in RPE cells [3]. Damaged mitochondria in aged RPE cells will consequently reduce the respiratory rate, ATP synthesis, and dysfunctional phagocytosis. The accumulative non-phagocytized OS debris may cause local inflammation and steer neovascularization and drusen deposit, the classical features of RP- and AMD-affected retina [4,5].

Although there are many causes of retinal degeneration, the final result is often retinal atrophy. Currently, limited therapy is available to treat retina atrophy. Treating RP patients with nutritional supplements can only delay RP’s progression. Anti-angiogenesis agents, such as Ranibizumab, Aflibercept, and Bevacizumab [6], are the first-line treatments for wet AMD by blocking the invasion of choroidal vascular endothelial cells. These anti-VEGF (vascular endothelial growth factor) therapies could reduce macular leakage and the regression of choroidal neovascularization. Still, they cannot rescue damaged retina from cell loss and atrophy.

Mitochondrial transplantation is an effective intervention to prevent the progression of acute inflammation such as cardiac infarct, cerebral stroke, and acute respiratory distress syndrome in experimental animals [7,8]. Interestingly, a recent paper demonstrated that intravitreal injection of mitochondria provided neuroprotection from optic nerve injury and modulation of oxidative metabolism [9]. Here, we aim to evaluate the transplanted allogeneic mitochondria as a new approach to treating retinal degeneration. We use Royal College of Surgeons (RCS) rats as the retinal degeneration animal model, which show inherited retinal degeneration and exhibit similar pathogenesis to RP [10,11]. The integrity of the retina layers in vivo and the functional transmission of nerve impulses were investigated for rescuing degenerative cells in the mitochondrial engrafted eyes.

## 2. Materials and Methods

### 2.1. Animals

The RCS/Kyo rats were obtained from the Institute of Laboratory Animals, Graduate School of Medicine, Kyoto University. These rats inherit a mutation of *MertK*, a receptor tyrosine kinase gene and have the impaired phagocytosis of RPE cells [12]. RCS rat is a classical disease model of RP in humans [10,11], and the rapid degeneration of the photoreceptor cells after 3 weeks of age is also a unique feature of macular degeneration [13] (http://www.anim.med.kyoto-u.ac.jp, accessed on 9 October 2022). Each group includes both male and female rats.

The experimental protocols and procedures were approved by the Institutional Animal Care and Use Committee of National Chung Hsing University (IACUC 104-008). We performed the animal experiments under Taiwan Animal Protection Act. Animals were housed and maintained at 25 °C under a light/dark cycle of 12 h environment. The food and water were provided ad libitum.

### 2.2. Mitochondria Extraction

The applied mitochondria were isolated from the livers of RCS rats. The rats show normal aspartate aminotransferase (AST) and alanine aminotransferase (ALT) activities, implying the integrity of liver mitochondria (https://rgd.mcw.edu, accessed on 9 October 2022, RGD ID, 1302660). The method of isolating mitochondria from the rat liver followed a general protocol [14] and showed high purity of isolated mitochondria in our previous study [15]. In brief, eight- to twelve-week-old RCS rats were sacrificed and systemically perfused with normal saline. The liver was washed with SEH buffer (0.25 M sucrose, 0.5 mM ethylene glycol tetraacetic acid[EGTA], 3 mM N-(2-Hydroxyethyl) piperazine-N’-ethanesulfonic acid [HEPES], pH 7.2; all from Sigma-Aldrich, Saint Lious, MI, USA) and homogenized by handheld homogenizer (IKA T10 basic, Homogenizer workcenter, IKA, Staufen, Germany). The 4 mL homogenates were centrifuged at 1000× *g* (Tomy Digital Biology, MX-301, Tokyo, Japan) for 15 min. The supernatants were applied to a discontinuous sucrose density gradient (30%, 40%, 55%) to separate the cell debris and the organelles by centrifuging at 35,000× *g* (Optima L-100K Ultracentrifuge, Beckman-Coulter, Brea, CA, USA) for 30 min. The mitochondrial fraction, between 40% and 55% sucrose density layer, was extracted and washed twice with 10 mL SEH at 13,000× *g*. The pellet of isolated mitochondria was resuspended in 2 mL SEH buffer and finally stored in Dulbecco’s Modified Eagle medium (DMEM, Gibco, Carlsbad, CA, USA) with 20 μL protease inhibitors (Sigma-Aldrich) with a 1:100 ratio. The quantity of mitochondria was measured by the total protein concentration of the mitochondrial pellets, determined by the Bradford method (Bio-Rad, Hercules, CA, USA). The process and storage of the isolated mitochondria were kept on the ice up to 5 h before the engraftment.

### 2.3. Mitochondria Transplantation and Tissue Analyses

RCS rats at 4 weeks old (N = 14) were anesthetized with isoflurane and received one intravitreal injection with a 33G Hamilton syringe per week. One eye received five injections during 5 weeks. In the control group, rats received 2 μL DMEM only. In the experiment group, rats received 50 μg or 100 μg mitochondria in 2 μL DMEM. The treated eyes were clear but showed low opacity at 1 day post-injection, especially in the 100 μg mitochondria group. The rats’ behavior was normal, and no severe adverse effect was observed in both control and experimental groups.

The eye samples were fixed with a buffer (formaldehyde:acetic acid:ethanol:distilled water = 2:10:35:53) and embedded in the paraffin by the end of the experiment. RCS rats were sacrificed at 9 weeks of age after the experiment. We commissioned the Animal disease diagnostic center (ADCC) at the college of veterinary medicine, National Chung Hsing University, to prepare tissue paraffin sections and hematoxylin and eosin (H&E) stains. A well-experienced pathologist diagnosed the severity of the RPE damage level between control and experimental groups at ADCC. H&E stain slices were obtained by serial cross-sectioning of the eye near the optical nerve. Eleven to twelve pictures were taken from each slice for retina assembly and measurement of retina thickness and ONL cell numbers (Supplementary Figure S1). The retina thickness was measured with the freeware of Meazure (C-thing, version 2.0.1).

### 2.4. Visual Function Determination

At 3, 5, and 9 weeks of age, RCS rats were anesthetized with an intramuscular injection of 20 mg/kg Zoletil (Virbac, Nice, France) and 50 μg/kg Dexdomitor (Zoetis, Kalamazoo, MI, USA). Before the examination of the visually evoked potential (VEP) and optical coherence tomography (OCT), each eye was treated with one drop of alcaine solution (0.5%, Alcon, Hünenberg, Switzerland) and tropicamide (0.5%, Akorn, Lake Forest, IL, USA) to dilate the pupil and help eye examination.

VEP signals were recorded with an Espion Visual Electrophysiology System E2 (Diagnosys LLC, Lowell, MA, USA) by implanting a recording electrode (OZ) on the occipital bone (7 mm behind the bregma and 3 mm lateral of the midline) and a reference electrode (FZ) into the scalp (12 mm behind the bregma), following previous protocols [16,17]. The ground electrode was placed into the tail. The patterned stimulus intensity was 3 cds/m^2^. The frequency of the flash stimulus was 1.02 Hz. After the VEP experiment, we added a drop of 1% methylcellulose on the rat eye and consequently conducted OCT with Phoenix Image-Guided OCT System (Phoenix Research Lab, Pleasanton, CA, USA) to measure and record the retina thickness of rats.

### 2.5. APRE-19 Cells Experiment

ARPE-19 cell is an immortalized human RPE cell line. Mitochondria used in in-vitro experiments were isolated from 2 × 10^7^ ARPE-19 cells. The adherent cells were isolated by trypsinization, and the collected cells were ground with SEH buffer on ice. After centrifugation at 1000× *g* for 15 min, the supernatants were collected and further centrifuged at 9000× *g* for 15 min (Tomy Digital Biology, MX-301, Tokyo, Japan). The pellets were resuspended in SEH buffer with a 1:100 diluted cocktail of protease inhibitors (Sigma-Aldrich). The mitochondrial mass was determined by Bradford method (Bio-Rad, Hercules, CA, USA).

Prior to the mitochondria treatment, 2 × 10^4^ ARPE-19 cells were grown in each well of a 24-well plate overnight. The cells were pretreated with indicated doses of H_2_O_2_ for two hours and then incubated with the exogenous mitochondria (0, 5 μg, 10 μg) for 24 h. We detected the fluorescence intensity of alamar blue reduction (BIO-RAD, Kidlington, UK) to reflect the mitochondrial activities of NADH, NADPH, as well as the cytochromes in the treated cells.

### 2.6. Statistic Analysis

Pair student *t*-test or ANOVA with bonferroni post hoc analysis were used to determine the significance of differences between the experimental groups. Graphics creation and statistical analysis in this study were conducted by using GraphPad Prism 5 (GraphPad, La Jolla, CA, USA).

## 3. Results

### 3.1. Retinal Degeneration in RCS Rats

**Figure 1:**
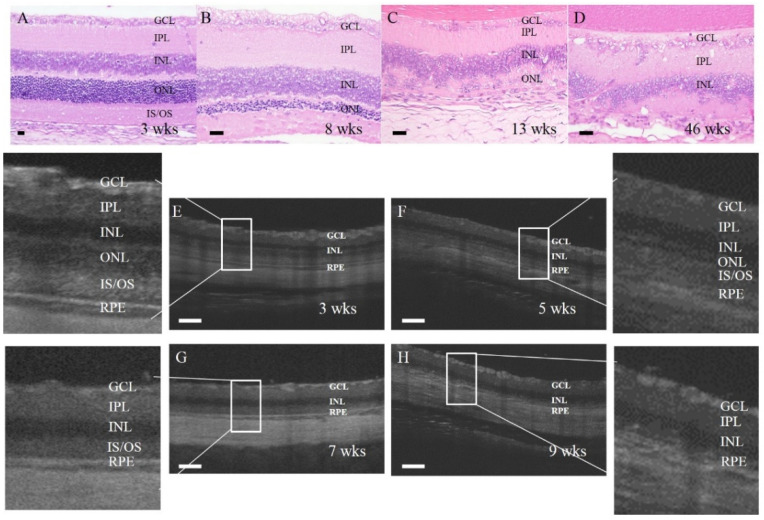
Retinal degeneration in RCS rats. (**A**–**D**) the histological structure of the rat retina at 3, 8, 13, and 46 weeks (wks) of age. (**E**–**H**) the representative photos of optical coherence tomography (OCT) at the fundus of RCS rats at 3, 5, 7, and 9 weeks of age. GCL: ganglion cell layer; IPL: inner plexiform layer; INL: inner nuclear layer; ONL: outer nuclear layer; IS/OS: inner segments/outer segments; RPE: retinal pigment epithelium. Scale bar in panel (**A**–**D**), 20 μm; scale bar in panel (**E**–**H**), 100 μm.

We first characterized the progress of retinal degeneration and pathological hallmarks in RCS rats. The retina at three weeks old was intact but showed decreased cell numbers in the outer nuclear layer (ONL) and the loss of inner and outer segments (IS/OS) of photoreceptors at eight weeks old. Severe cell loss and the drastically diminished thickness of outer and inner cell layers were noticed at 13 and 46 weeks old by the histological examination near the optic disc and in vivo OCT tracking (Figure 1A–D). The continuous in vivo recording by OCT illustrated that the retina thickness sharply decreased from 207 μm to 133 μm between four and eight weeks old (18.5 μm/week reduction). The thickness became 109 μm till the age of 11 weeks (8 μm/week reduction), with the feature of missing outer nuclear layer and IS/OS (Figure 1E–H).

**Figure 2:**
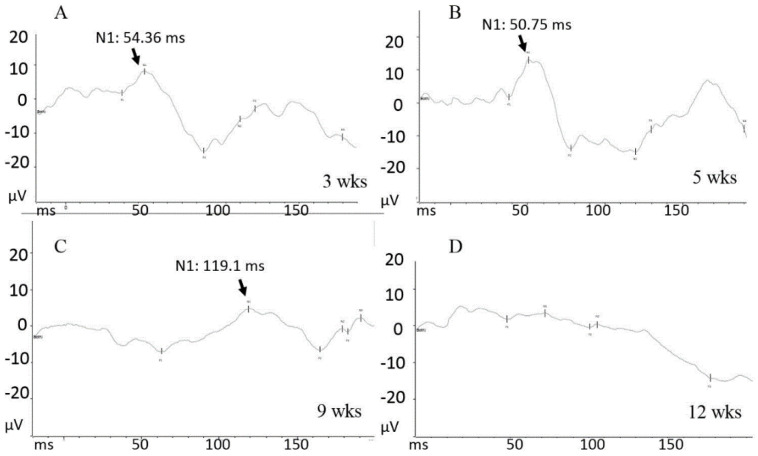
The records of visually evoked potential (VEP) at 3 (**A**), 5 (**B**), 9 (**C**), and 12 (**D**) weeks (wks) of RCS rats. X axis, ms: mini-second. Y axis, μV, micro-voltage. The N1 latency, representing the time of photo-transduction rate from eye to visual cortex, is indicated by arrow.

The nerve conduction test by VEP revealed that the three-week-old retinae of RCS rats exhibited standard physiological VEP profiles. The classical wave amplitude and N1 latency were detected around 50 mini-second (ms). In contrast, nine-week-olds showed a drastic amplitude reduction and prolonged N1 latency. These results suggest that the age period between five and nine weeks initiates the early pathogenesis process and should be a proper window to evaluate potential treatment for intervening the retina degeneration in RCS rats (Figure 2A–D).

### 3.2. Mitochondrial Transplantation Moderately Ameliorated Retina Degeneration

Due to the small size and instability of the mitochondria, validation of surgical procedures and tracing the engrafted mitochondria in vivo for a long term are challenging [9,18,19]. To validate the success of intravitreal injection in rats, homogenous fluorescent beads (7.5 μm, Becton-Dickinson) were applied to imitate mitochondria initially. Images of computational tomography (CT) and fluorescent spectrometry revealed that the beads were exclusively accumulated in the vitreous bodies, supporting the reliability of the experimental surgical procedure of mitochondrial transplantation (Supplementary Figure S2).

**Figure 3:**
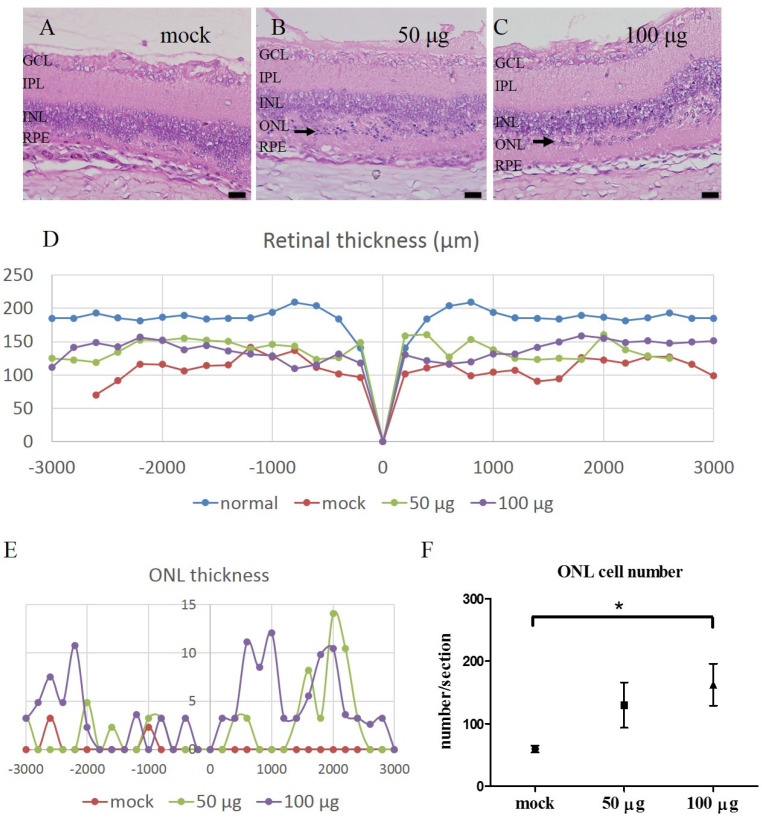
(**A**–**C**) The retina layer thickness in the central and peripheral optic disc regions of the mitochondria-treated eyes. The representative retina histology with H&E stain, located at a 1 mm distance from the optic disc, in mock-treated RCS rats and mitochondria-treated groups. (**D**) The horizontal thickness distributions of the retina near the optic disc in mock-treated RCS rats and mitochondria-treated groups, summarized from the results of serial sections of eyes. Normal: the retina of 3-wks old RCS rats. (**E**) The ONL thickness in panel (**D**). (**F**) The average cell numbers of ONL in panel (**E**), analyzed from mock- (N = 6) and mitochondria-treated eyes (50 and 100 μg, N = 8 for each group). Mock vs. 50 μg, *p* = 0.105; mock vs. 100 μg, * *p* = 0.018, paired *t*-test). The scale bar: 20 μm. GCL: ganglion cell layer; IPL: inner plexiform layer; INL: inner nuclear layer; ONL: outer nuclear layer; RPE: retinal pigment epithelium.

Our previous studies have shown that, following our established protocol, the isolated mitochondria from livers were morphologically intact, high-purity, and had functional respiratory activities [15]. In this study, 50 μg and 100 μg allogeneic mitochondria were transplanted into the vitreous bodies of RCS rats at four weeks old, and the integrity of the retina was examined at nine weeks old. The eyes (mock, N = 6; 50 μg group, N = 8; 100 μg group, N = 8) were collected and subjected to histological examination. H&E staining indicated that engrafted mitochondria rescued the loss of the outer cell layer and also the layers of IS/OS (Figure 3A–C). The trend of the therapeutic effect of mitochondria was illustrated in Figure 3D,E, showing the rescue of both the retina layer thickness and the outer cell layer thickness from the optic disc’s central and peripheral loci (3 mm diameter) from one representative eye of each group. We further counted the ONL cell numbers within the same range of Figure 3D of all eyes (mock, N = 6; 50 μg group, N = 8; 100 μg group, N = 8). The average cell numbers of the transverse section in mock, 50 μg, and 100 μg mitochondria-treated eyes were 59.5 ± 5.3, 129.8 ± 36.0, and 162.4 ± 33.5, respectively (mock vs. 100 μg group, student *t*-test, *p* < 0.05) (Figure 3F). These H&E results indicated that both 50 μg and 100 μg mitochondrial treatment at nine weeks old exhibited moderate potency in preventing retina degeneration (Figure 3).

### 3.3. OCT Recording of the Retina after Mitochondrial Transplantation

**Figure 4:**
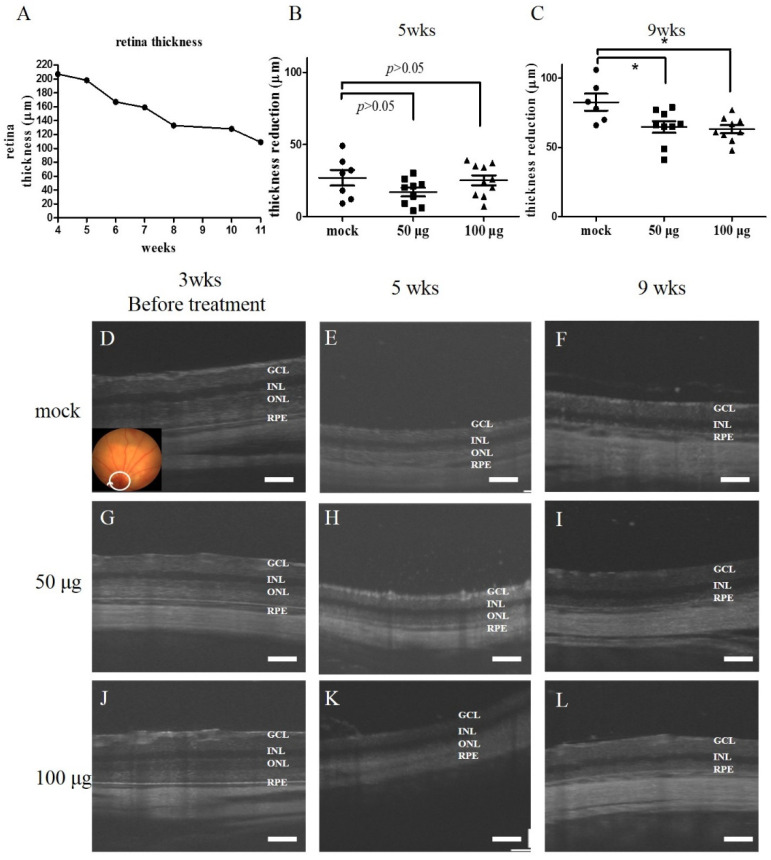
The rescue of the progressive retina degeneration after mitochondrial transplantation in RCS rats. (**A**) one of RCS rat retina thickness continuous recording by OCT from 4 to 11 weeks of age. The losses of the retina thickness at 5 weeks (wks) of age (**B**), and at 9 weeks of age (**C**) were compared between the mock-treated control and the mitochondria-treated groups (50 μg and 100 μg). The value of thickness reduction was the reduced length (μm) of the retina relative to the individually measured retina thickness at 3 weeks of age. * *p* < 0.05. (**D**–**L**) Representative OCT images of the control group and mitochondria-treated group of the rats at 3, 5, and 9 weeks of age. Scale bar in panel (**D**–**L**), 100 μm. GCL: ganglion cell layer; INL: inner nuclear layer; ONL: outer nuclear layer; RPE: retinal pigment epithelium.

In addition to the histological examination, we provided in vivo degeneration data of mock-treated control and mitochondria-treated groups by examining optical coherence tomography (OCT) (Figure 4) (mock, N = 6; 50 μg group, N = 9; 100 μg group, N = 9). We found that the thickness loss at five weeks old was slightly attenuated after 50 μg mitochondrial transplantation (16.9 ± 3 μm vs. 26.9 ± 5.5 μm) (*p*-value, 0.11) (Figure 4A,B). This anti-degeneration activity was sustained at nine weeks old in the 50 μg and 100 μg groups (64.8 ± 4 μm and 63.1 ± 3 μm, respectively) compared to that of the mock-treated control (82.7 ± 6 μm) (*p*-value, 0.03 and 0.01, respectively) (Figure 4C) (summarized from Figure 4D–L. This result was consistent with the statistical results of the retina histological sections in Figure 3D–F.

### 3.4. VEP Recording of the Retina after Mitochondria Transplantation

**Figure 5:**
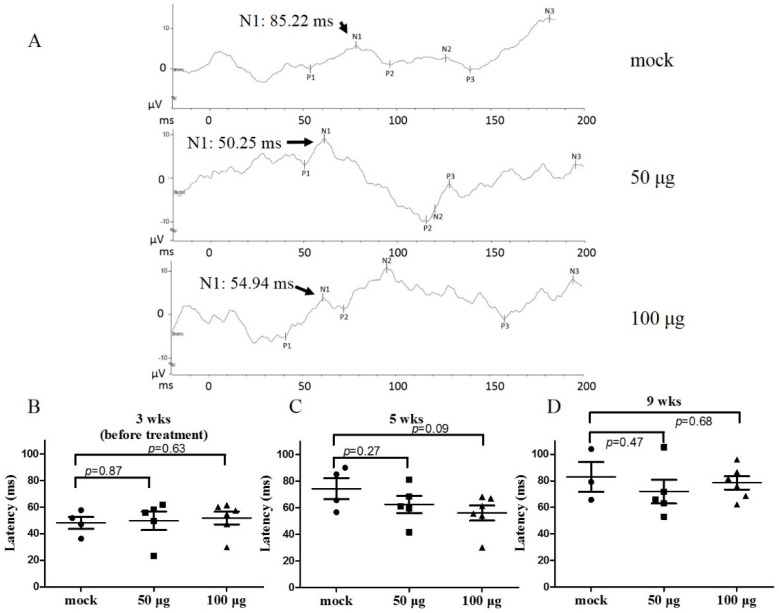
VEP recording of RCS rats after mitochondrial transplantation. (**A**) Representative VEP waveforms in the control rat and mitochondria-treated groups at 5 weeks of age. (**B**–**D**) The summary of the timing of VEP N1 latency in the control rat and mitochondria-treated groups at 3 (**B**), 5 (**C**), 9 weeks (**D**) of age. VEP: visual evoked potential.

The time of afferent nerve impulses from the eyes to the cortex, determined by VEP, was investigated in this study to reflect the retina’s electrical activity. At the ages of three (prior to treatment) and five weeks (one-week post-treatment), the averaged N1 latencies in mock-treated RCS rats were 48.25 ± 4.5 and 74.4 ± 7.9 ms, respectively (Figure 5B and Figure 5C lane 1, each group N ≥ 4). After mitochondrial transplantation at five weeks old, VEP recording showed 62.4 ± 6.4 ms and 56.3 ± 5.7 ms N1 latency in 50 μg and 100 μg mitochondria groups, respectively (Figure 5A,C). Although there was no significant difference between the results of each group (Figure 5C), the prolonged N1 latency was attenuated by mitochondrial transplantation with a dose-dependent trend (12.0 ms and 18.1 ms, after 50 μg and 100 μg mitochondria treatment, respectively).

We continued the mitochondrial transplantation weekly for five weeks and observed the VEP at nine weeks old. All VEP data exhibited a non-classical profile and prolonged N1 latency over 70 ms. No significant difference was detected among the recording results of each group (Figure 5D). These results suggest that at the late stages of retina degeneration, the damage is too severe to show the functional difference, or VEP test is not sensitive to evaluate functional recovery in this study [20].

## 4. Discussion

A recent clinical trial showed that mitochondrial injection might be a promising approach to prevent the death of cardiomyocytes and restore heart functions in pediatric patients [21]. Animal experiments also reveal that local mitochondrial transplantation successfully attenuates acute tissue injuries and preserves physiological activities in damaged heart, liver, lung, and nerve systems [7,22,23]. In this pilot experiment, we provide the first evidence that delivered mitochondria in the vitreous bodies could moderately attenuate photoreceptor degeneration in rats with inherited retina degeneration. We demonstrated the ONL and IS/OS reservation by OCT examination and the joint restored function by VEP activity.

It is critical to illustrate the mechanism of action (MOA) that underlies the protective effects before human mitochondrial transplantation is applied. Although many studies reproduce the therapeutic effects of engrafted mitochondria, it is still challenging to trace the systemically or locally delivered mitochondria in targeted tissues [24]. This limitation prevents the evaluation of pharmacokinetics (PK) and pharmacodynamics (PD) of mitochondrial therapy. For instance, injected mitochondria in hearts were dispersed in extracellular mesenchyme, or phagocytized by cardiomyocytes. Infiltrated immune cells can uptake the mitochondria and transfer them to the circulating reticuloendothelial system (RES) and microcapillary system [7]. Moreover, the injection site milieu, the injury severity, and the RES clearing activity can affect the biodistribution profile.

These dynamic biodistributions of the transferred mitochondria complicate the exploration of the underlying MOA. The anti-degeneration of engrafted mitochondria may be mediated by rescuing the death of target cells via released metabolites or anti-oxidative activity of mitochondria *per se*. The necessity and the efficacy of the internalization of the mitochondria into target cells should be validated [25]. It is also plausible that delivered mitochondria modulate the activities of infiltrated monocytes, macrophage, or endothelial progenitor cells and consequently attenuate local inflammatory reaction and destructive cytokine storm [26].

Deficiency of *MertK*-related phagocytosis results in defective elimination of abnormal mitochondria, reduced respiration capacity, and even the activation of the inflammasome in other tissues [27,28]. In the RCS rats, the defective *MertK* gene disrupts RPE cells’ phagocytosis activity, resulting in impaired elimination of outer segment fragments of photoreceptors, photoreceptor degeneration, and retina atrophy. In this study, we provide a simplified in vivo model to investigate the mechanism of mitochondria-mediated treatment. Delivered mitochondria were restricted in a closed chamber of the affected eye, and the in vivo therapeutic effect can be quantified by the OCT recording. The inner nuclear layer (INL) of RCS rats was generally intact, suggesting that the activity of INL cells, such as müller glial cells and amacrine cells, and integrity of the inner limiting membrane were sustained. It is practical to trace labeled mitochondria periodically and elucidate the diffusion rates or active transportation for the mitochondrial distribution in retina layers through tangential sections of the engrafted retina [29].

In our model, the therapeutic effect of mitochondrial transplantation was first observed at five weeks old. This improved visual function and reduced tissue degeneration might be related to the rescued mitochondrial activities in photoreceptors, the increased growth factor secretion by Mueller glial cells or the restored phagocytosis by RPE cells. The transplanted mitochondria might increase the mtDNA contents or fuse with the abnormal mitochondria of targeted cells to rescue its function or prevent the formation of inflammasomes in retinal cells [15]. In addition, neuroprotective mitochondria-derived peptides may play some roles in mitochondria-mediated retina protection [30]. Moreover, whether transplanted mitochondria directly or indirectly prevent the apoptosis or necroptosis of the photoreceptor cells needs to be addressed. According to the histopathological evidence, it is more plausible that transplanted mitochondria mainly prevent the necroptosis at the late stage of the retina degeneration, but not the early-stage apoptosis [13]. These issues are critical to distinguishing the role of transplanted mitochondria in coping against photoreceptor cell death [22]. We have in vitro evidence that treating APRE-19 cells with exogenous mitochondria can restore the mitochondrial biochemical activities (Figure S3), suggesting that the engrafted mitochondria might rescue the mitochondrial functions and damaged phagocytosis of RPE cells in RCS rats.

## 5. Conclusions

In this study, we demonstrate a new ocular animal model for the study of mitochondria-mediated therapy. Several critical issues for the mitochondria-mediated therapy could be investigated in this model, such as the in vivo shelf-life of mitochondria, the biodistribution, the PK/PD information, and the dose-escalation relationship on cytotoxic and therapeutic effects without the interference of immune cells and vascular endothelial cell invasion. The necessity, efficacy, and potential mechanism of the internalization of the mitochondria into target cells can also be validated and traced by high-resolution images and immuno-histological staining. With these consequential investigations, we may glimpse the potential of mitochondria-mediated therapy in ocular disorders.

## Data Availability

The data that support the findings of this study are available from Hong Lin Su but restrictions apply to the availability of these data, which were used under license for the current study, and so are not publicly available. Data are however available from the authors upon request and with the permission of Hong Lin Su.

## Supplementary Materials

The following supporting information can be downloaded at: https://www.mdpi.com/article/10.3390/biomedicines10112883/s1, Figure S1: The RCS retina with high resolution, assembled from 11-12 serial photos for measuring the retina thickness and ONL cell numbers; Figure S2: The distribution of engrafted fluorescent beads in the eyes of RCS rats after intravitreal injection 1 day; Figure S3: Dose-effects of exogenous mitochondria on rescuing the Alamar blue reduction ability and cell numbers of H_2_O_2_-treated APRE-19 cells.

## References

[1] Pagano G., Pallardo F.V., Lyakhovich A., Tiano L., Trifuoggi M. (2021). Mitigating the pro-oxidant state and melanogenesis of Retinitis pigmentosa: By counteracting mitochondrial dysfunction. Cell. Mol. Life Sci..

[2] Ferrington D.A., Fisher C.R., Kowluru R.A. (2020). Mitochondrial Defects Drive Degenerative Retinal Diseases. Trends Mol. Med..

[3] Stone J., van Driel D., Valter K., Rees S., Provis J. (2008). The locations of mitochondria in mammalian photoreceptors: Relation to retinal vasculature. Brain Res..

[4] Blasiak J., Glowacki S., Kauppinen A., Kaarniranta K. (2013). Mitochondrial and nuclear DNA damage and repair in age-related macular degeneration. Int. J. Mol. Sci..

[5] Terluk M.R., Kapphahn R.J., Soukup L.M., Gong H., Gallardo C., Montezuma S.R., Ferrington D.A. (2015). Investigating mitochondria as a target for treating age-related macular degeneration. J. Neurosci..

[6] Hernandez-Zimbron L.F., Zamora-Alvarado R., Ochoa-De la Paz L., Velez-Montoya R., Zenteno E., Gulias-Canizo R., Quiroz-Mercado H., Gonzalez-Salinas R. (2018). Age-Related Macular Degeneration: New Paradigms for Treatment and Management of AMD. Oxid. Med. Cell. Longev..

[7] McCully J.D., Levitsky S., Del Nido P.J., Cowan D.B. (2016). Mitochondrial transplantation for therapeutic use. Clin. Transl. Med..

[8] McCully J.D., Cowan D.B., Pacak C.A., Toumpoulis I.K., Dayalan H., Levitsky S. (2009). Injection of isolated mitochondria during early reperfusion for cardioprotection. Am. J. Physiol.-Heart Circ. Physiol..

[9] Nascimento-dos-Santos G., de-Souza-Ferreira E., Lani R., Faria C.C., Araújo V.G., Teixeira-Pinheiro L.C., Vasconcelos T., Gonçalo T., Santiago M.F., Linden R. (2020). Neuroprotection from optic nerve injury and modulation of oxidative metabolism by transplantation of active mitochondria to the retina. Biochim. Biophys. Acta (BBA)—Mol. Basis Dis..

[10] Conlon T.J., Deng W.T., Erger K., Cossette T., Pang J.J., Ryals R., Clement N., Cleaver B., McDoom I., Boye S.E. (2013). Preclinical potency and safety studies of an AAV2-mediated gene therapy vector for the treatment of MERTK associated retinitis pigmentosa. Hum. Gene Ther. Clin. Dev..

[11] Deng W.T., Dinculescu A., Li Q., Boye S.L., Li J., Gorbatyuk M.S., Pang J., Chiodo V.A., Matthes M.T., Yasumura D. (2012). Tyrosine-mutant AAV8 delivery of human MERTK provides long-term retinal preservation in RCS rats. Investig. Ophthalmol. Vis. Sci..

[12] Burstyn-Cohen T., Lew E.D., Traves P.G., Burrola P.G., Hash J.C., Lemke G. (2012). Genetic dissection of TAM receptor-ligand interaction in retinal pigment epithelial cell phagocytosis. Neuron.

[13] Mizukoshi S., Nakazawa M., Sato K., Ozaki T., Metoki T., Ishiguro S.-i. (2010). Activation of mitochondrial calpain and release of apoptosis-inducing factor from mitochondria in RCS rat retinal degeneration. Exp. Eye Res..

[14] Graham J.M. (1999). Isolation of Mitochondria from Tissues and Cells by Differential Centrifugation. Curr. Protoc. Cell Biol..

[15] Chang J.C., Hoel F., Liu K.H., Wei Y.H., Cheng F.C., Kuo S.J., Tronstad K.J., Liu C.S. (2017). Peptide-mediated delivery of donor mitochondria improves mitochondrial function and cell viability in human cybrid cells with the MELAS A3243G mutation. Sci. Rep..

[16] Tomita H., Sugano E., Yawo H., Ishizuka T., Isago H., Narikawa S., Kugler S., Tamai M. (2007). Restoration of visual response in aged dystrophic RCS rats using AAV-mediated channelopsin-2 gene transfer. Investig. Ophthalmol. Vis. Sci..

[17] Papathanasiou E.S., Peachey N.S., Goto Y., Neafsey E.J., Castro A.J., Kartje G.L. (2006). Visual cortical plasticity following unilateral sensorimotor cortical lesions in the neonatal rat. Exp. Neurol..

[18] Masuzawa A., Black K.M., Pacak C.A., Ericsson M., Barnett R.J., Drumm C., Seth P., Bloch D.B., Levitsky S., Cowan D.B. (2013). Transplantation of autologously derived mitochondria protects the heart from ischemia-reperfusion injury. Am. J. Physiol.-Heart Circ. Physiol..

[19] Cowan D.B., Yao R., Akurathi V., Snay E.R., Thedsanamoorthy J.K., Zurakowski D., Ericsson M., Friehs I., Wu Y., Levitsky S. (2016). Intracoronary Delivery of Mitochondria to the Ischemic Heart for Cardioprotection. PLOS ONE.

[20] Bass S.J., Sherman J., Bodis-Wollner I., Nath S. (1985). Visual evoked potentials in macular disease. Investig. Ophthalmol. Vis. Sci..

[21] Emani S.M., McCully J.D. (2018). Mitochondrial transplantation: Applications for pediatric patients with congenital heart disease. Transl. Pediatr..

[22] Huang P.J., Kuo C.C., Lee H.C., Shen C.I., Cheng F.C., Wu S.F., Chang J.C., Pan H.C., Lin S.Z., Liu C.S. (2016). Transferring Xenogenic Mitochondria Provides Neural Protection Against Ischemic Stress in Ischemic Rat Brains. Cell Transpl..

[23] Roushandeh A.M., Kuwahara Y., Roudkenar M.H. (2019). Mitochondrial transplantation as a potential and novel master key for treatment of various incurable diseases. Cytotechnology.

[24] McWilliams T.G., Ganley I.G. (2016). Life in lights: Tracking mitochondrial delivery to lysosomes in vivo. Autophagy.

[25] Caicedo A., Aponte P.M., Cabrera F., Hidalgo C., Khoury M. (2017). Artificial Mitochondria Transfer: Current Challenges, Advances, and Future Applications. Stem Cells Int..

[26] Jackson M.V., Morrison T.J., Doherty D.F., McAuley D.F., Matthay M.A., Kissenpfennig A., O’Kane C.M., Krasnodembskaya A.D. (2016). Mitochondrial Transfer via Tunneling Nanotubes is an Important Mechanism by Which Mesenchymal Stem Cells Enhance Macrophage Phagocytosis in the In Vitro and In Vivo Models of ARDS. Stem Cells.

[27] Nicolas-Avila J.A., Lechuga-Vieco A.V., Esteban-Martinez L., Sanchez-Diaz M., Diaz-Garcia E., Santiago D.J., Rubio-Ponce A., Li J.L., Balachander A., Quintana J.A. (2020). A Network of Macrophages Supports Mitochondrial Homeostasis in the Heart. Cell.

[28] Peeters M.J.W., Dulkeviciute D., Draghi A., Ritter C., Rahbech A., Skadborg S.K., Seremet T., Carnaz Simoes A.M., Martinenaite E., Halldorsdottir H.R. (2019). MERTK Acts as a Costimulatory Receptor on Human CD8(+) T Cells. Cancer Immunol. Res..

[29] Strissel K.J., Lishko P.V., Trieu L.H., Kennedy M.J., Hurley J.B., Arshavsky V.Y. (2005). Recoverin undergoes light-dependent intracellular translocation in rod photoreceptors. J. Biol. Chem..

[30] Yang Y., Gao H., Zhou H., Liu Q., Qi Z., Zhang Y., Zhang J. (2019). The role of mitochondria-derived peptides in cardiovascular disease: Recent updates. Biomed. Pharmacother..

