# Supplementary material for: Mitochondrial Transplantation Moderately Ameliorates Retinal Degeneration in Royal College of Surgeons Rats
Source: Biomedicines. 2022 Nov 10;10(11):2883. doi: 10.3390/biomedicines10112883 (PMC9687640; doi:10.3390/biomedicines10112883)
Supplement: Supplementary file 1 [file biomedicines-10-02883-s001.zip › biomedicines-1958258-supplementary.pdf]

Supplementary Figure Legends

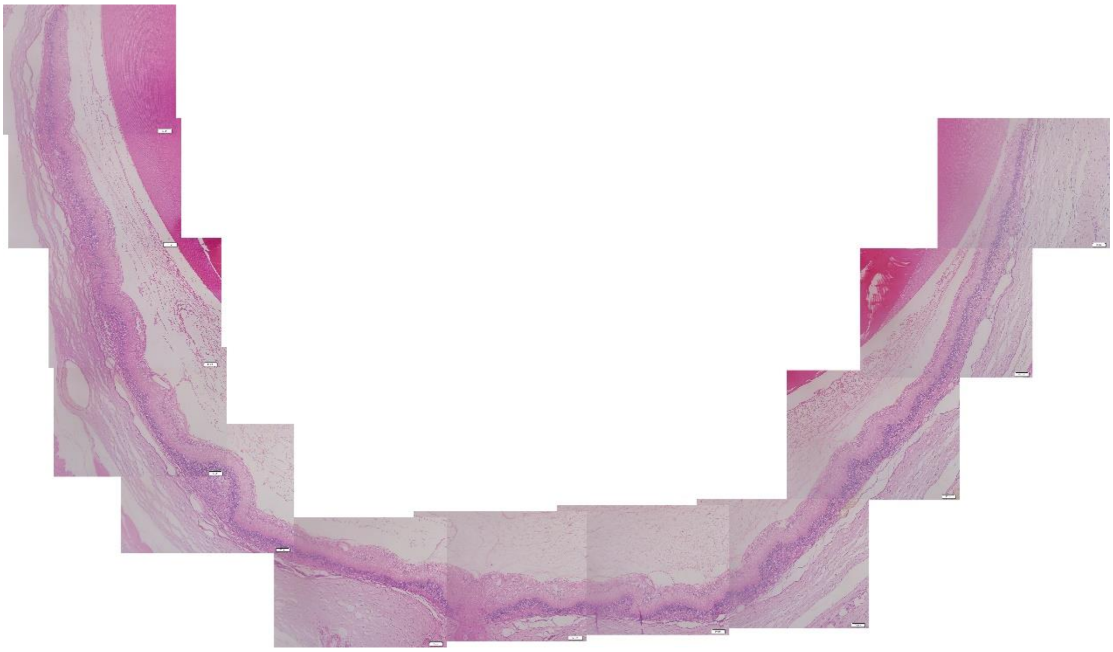

Figure S1. The RCS retina with high resolution, assembled from 11-12 serial photos for measuring the retina thickness and ONL cell numbers. The white bar in a photo, 50 μm.

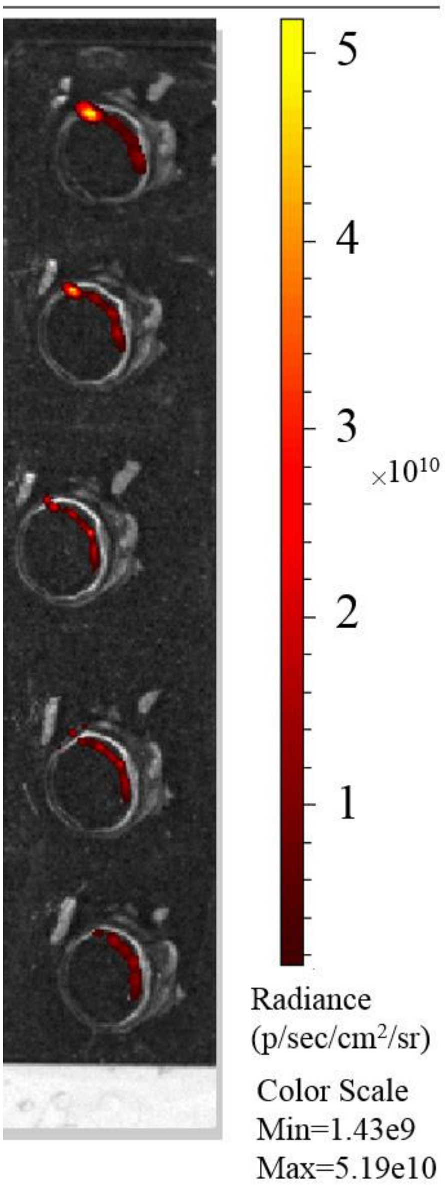

Figure S2. The distribution of engrafted fluorescent beads in the eyes of RCS rats after intravitreal injection 1 day.

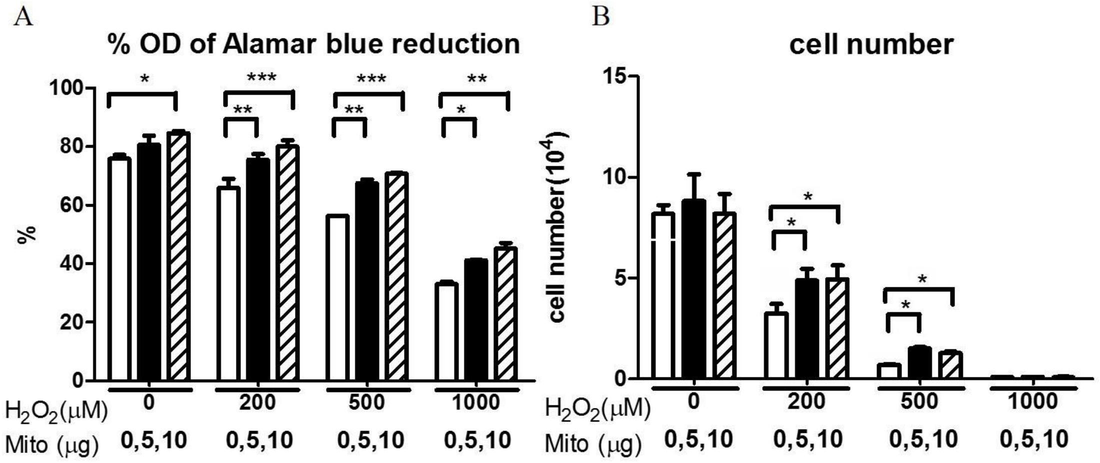

Figure S3. Dose-effects of exogenous mitochondria on rescuing the (A) Alamar blue reduction ability and (B) cell numbers of H_2O_2-treated APRE-19 cells. The spectrophotometry calculation of the ratio of Alamar blue reduction follows the instrument protocol (Bio-Rad Laboratories, CA, USA). *, p<0.05, **, p<0.01, ***, p<0.001, two-way ANOVA.
