# Supplementary figures and images for: Mitochondrial Transplantation Moderately Ameliorates Retinal Degeneration in Royal College of Surgeons Rats
Source: Biomedicines. 2022 Nov 10;10(11):2883. doi: 10.3390/biomedicines10112883 (PMC9687640; doi:10.3390/biomedicines10112883)

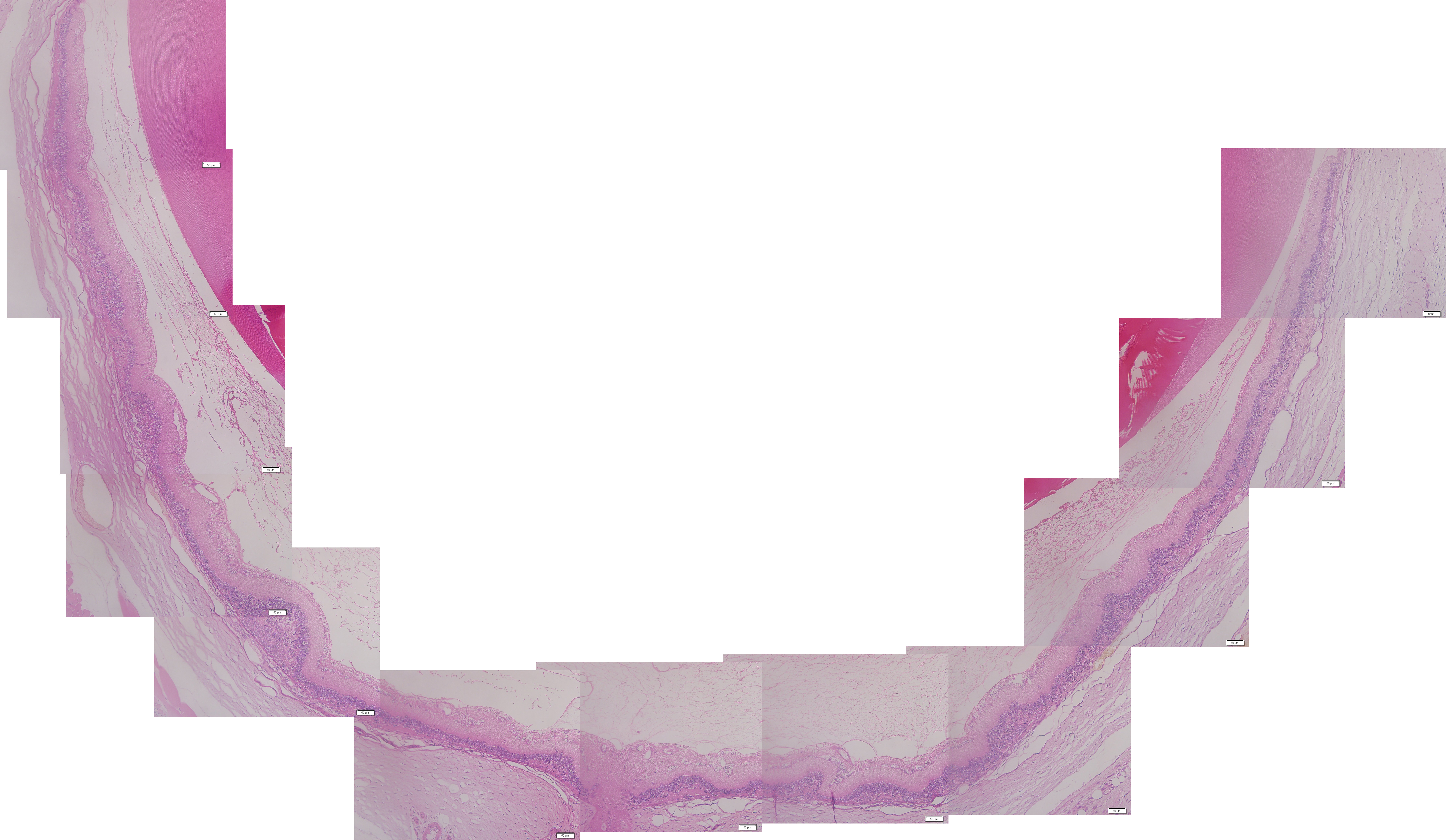

Supplement: Supplementary file 1 [file biomedicines-10-02883-s001.zip › Figure S1.jpg]

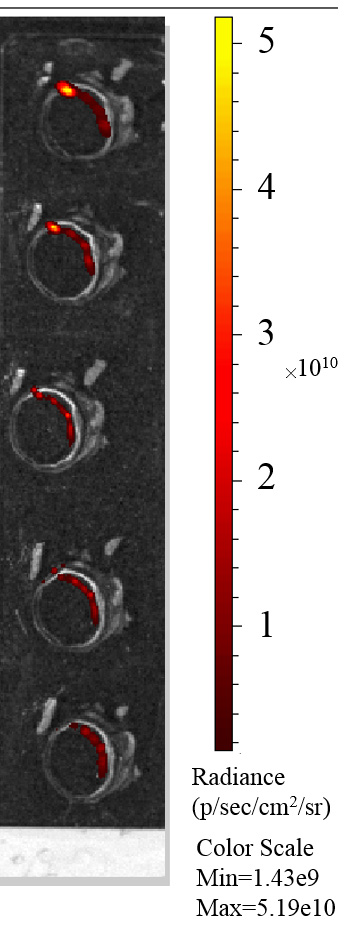

Supplement: Supplementary file 1 [file biomedicines-10-02883-s001.zip › Figure S2 v2.jpg]

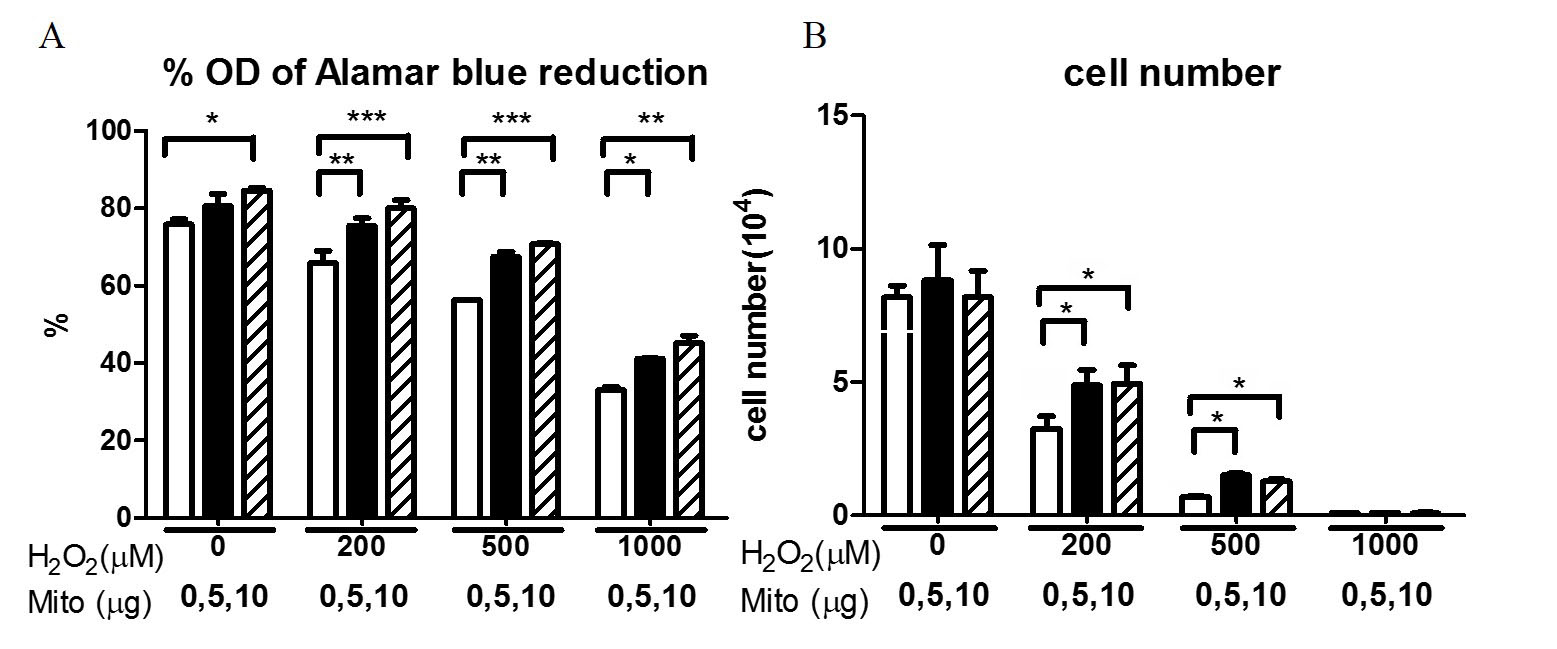

Supplement: Supplementary file 1 [file biomedicines-10-02883-s001.zip › Figure S3 v2.jpg]
